# Therapeutic effect of proprioceptive dolphin assisted activities on health-related quality of life and muscle tension, biomechanical and viscoelastic properties in major depressive disorder adults: case analysis

**DOI:** 10.3389/fnhum.2024.1487293

**Published:** 2025-01-14

**Authors:** Brigita Kreivinienė, Laura Šaltytė-Vaisiauskė, Sonata Mačiulskytė

**Affiliations:** ^1^Faculty of Health Sciences, Klaipėda University, Klaipėda, Lithuania; ^2^Faculty of Marine Technologies and Natural Sciences, Klaipėda University, Klaipėda, Lithuania

**Keywords:** depression, dolphin assisted activities, quality of life, tension, biomechanical and viscoelastic properties

## Abstract

**Introduction:**

The case analysis method is widely used in evaluating dolphin assisted activities due to the lack of research participants. Despite other present diagnostic features adults with major depressive disorder experience psychomotor changes, dysphoria, anhedonia, impaired concentration, and suicidal thoughts. Increasing studies assess the positive effect of proprioceptive exercises in various depressive cases.

**Methods:**

14 adults diagnosed major depressive disorder included in this case study between September 2022 to April 2024. A two-week proprioceptive dolphin assisted activity program was applied for each with a two-day break after half applied activities, in total 10 activities for each were organised. Proprioceptive dolphin assisted activities varied from heavy muscle work to muscles and joints pressure which was held in a special therapeutic pool, where adults wore wetsuits. The SF—36 scale was used, with levels of QoL compared among: (a) before dolphin assisted activities, (b) after two-week proprioceptive dolphin assisted activities, (c) in a year after two-week proprioceptive dolphin assisted activities. The MyotonPro portable device was used for measuring muscle tension, biomechanical and viscoelastic properties each day of participation: (a) before dolphin assisted activities and (b) after dolphin assisted activities. The MyotonPro was applied to measure the upper (descending) part of the trapezius muscle, lateral part of the deltoids muscle, middle point of the flexor carpi radialis muscle, middle point of the long head of the biceps brachii muscle, the gastrocnemius muscle (plural gastrocnemii), and quadriceps femoris muscle.

**Results:**

The measuring results of SF—36 scale showed statistically significant changes in 5 subscales out of 8 (Friedman’s test *p*-value less than 0.05) and in one more scale a partially significant change (*p* < 0.1). The physical activity that increased after dolphin therapy (from 68.5 to 85) remained the same in one year. There were no statistically significant changes in role limitations due to physical health. However, role limitations due to emotional problems and partially significant changes were recorded, the situation slightly worsened a year later, but there was no statistically significant deterioration. The non-parametric Wilcoxon test was used to compare two dependent samples measured by the MyotonPro. Although the improvement was recorded in almost all muscle groups, statistically significant changes were observed only in trapezius muscle md p right, measuring stiffness, relaxation and creep; trapezius md p left measuring frequency, stiffness, relaxation and decrement; and deltoids muscle left, measuring frequency, stiffness and relaxation, i.e., the *p*-values of Wilcoxon test are less than 0.05.

**Conclusion:**

Two-weeks of proprioceptive dolphin assisted activities for adults with major depression have statistically significant long-term change in better physical parameters. Emotional betterment parameters were less stable. The quick reactivity of trapezius muscle revealed that dolphin assisted activities acts as stress relief, and deltoids muscle as relief of negative emotions, such as fear and anger.

## 1 Introduction

Major depressive disorder (MDD) is a debilitating disease that is characterised by depressed mood, diminished interests, impaired cognitive function and vegetative symptoms, such as disturbed sleep or appetite (Otte et al., 2016). In 2008 MDD was ranked by WHO as the third course of burden diseases however in 2030 it is expected to be in the first place (Malhi and Mann, 2018). MDD is diagnosed approximately from 6 to 12 months of illness prevalence as far as a primary issue is often manifested to physicians as somatic pains streaming from depression (Bains and Abdijadid, 2023). As far disease is caused by many social, physical and psychological factors, worldwide practice document combination treatment as most effective (Cuijpers et al., 2009).

This article focuses of depression and physical proprioceptive activity as having a reciprocal inverse relationship (Kumar and Jim, 2010; Edwards and Loprinzi, 2016; Blough and Loprinzi, 2018). One of the vast-manifesting depression symptoms is an increase in sedentary lifestyle. Due to a constant sedentary lifestyle, it is assumed that depression influences reduced motor functioning and increased perception of physical disability. Physical disability leads to reducing the quality of life (QoL) on average to severe (Daneshvar et al., 2019). There are many studies evaluating the effects of exercise on physical functioning, and still few studies evaluating its effects on mental functioning. However, recently, there has been an increasing number of studies evaluating exercise as an additional or direct method for managing depressive symptoms (Edwards and Loprinzi, 2016; Blough and Loprinzi, 2018; Abdelbasset and Alqahtani, 2019; Abdelbasset et al., 2019; Abdelbasset et al., 2020). A randomized controlled trial reported by Edwards and Loprinzi (2016) and Blough and Loprinzi (2018) showed that sedentary behavior and even reduced habitual physical activity increased the risk of psychological health deprivation even in healthy subjects. Abdelbasset and colleagues published a series of reports investigating the effect of proprioceptive exercise training on depression and other psychological conditions in various populations such as patients with cardiovascular conditions (2019) and diabetic neuropathy (2020). They applied various length moderate-intensity continuous aerobic exercise sessions (from 8 to 12 weeks), in all trials they document the safety, suitability and effectiveness of a low- to moderate-intensity aerobic exercise program for reducing depression severity. Abdelbasset et al. (2019) study also highlights the effectiveness of the proprioceptive program when used in conjunction with standard medical treatment. The authors (Edwards and Loprinzi, 2016; Blough and Loprinzi, 2018; Abdelbasset and Alqahtani, 2019; Abdelbasset et al., 2019; Abdelbasset et al., 2020) recommend to include regular proprioceptive exercise training to prevent mental health decline in various populations, either they have heath issues or not.

A vast meta-analysis (see Vancampfort et al., 2017) gave a substantial body of evidence that sedentary behavior and physical activity levels correlates in people with severe mental illness therefore, it leads to social isolation, unemployment, higher body mass, and secondary physical issues, such as cardiovascular diseases and earlier mortality. Moreover, Westgaard (1999) manifested that experienced psychological or physical stressor may induce low levels of muscle tension forcing small motor units continuing active without rest and cause potential somatic pain development.

Physical therapy was shown to have positive influence on QoL and perceived well-being in a wide range of patient populations including mental disorders (Kumar and Jim, 2010). It is not yet clear what is the mechanism of the effect of physical exercise on the mental state, but a list of assumptions and hypotheses is put forward. During exercise, the level of the hormone endorphin in the blood increases (Daneshvar et al., 2019). Endorphin is an antidepressant hormone that creates vitality in the body. The most effective way to increase the level of endorphins in the body is to have a regular and exciting exercise (Cleare et al., 2015). Dolphin assisted therapy (DAT) has several powerful characteristics of excitement and vitality: animal involvement and water. Furthermore, dolphin assisted therapy that we targeted as an aquatic proprioceptive neuromuscular facilitation program shows even more significant changes in depression and improves quality of life (Lee and Hwang, 2019). The positive and non-demanding environment in such type of therapy is documented as having positive social interaction effect in autism (Griffioen et al., 2019), also investigations in cerebral paralysis case emerged as promising complementary therapy method where EEG indicated that dolphin-augmented sounds can activate separate brain regions in particular modes (Moreno Escobar et al., 2024). Despite existing criticism on DAT programs (Marino and Lilienfeld, 1998, 2007; Marino, 2014) numerous studies manifest of sensory, cognitive and bodily integration improvement assisted by the dolphin (Taylor and Carter, 2018). Majority of DAT studies focus on social, psychological, cognitive and emotional betterment of patients and their caregivers (see Stumpf and Breitenbach, 2014; Perttula and Kreivinienė, 2014; Nathanson et al., 1997; Lukina, 1999; Antonioli and Reveley, 2005; Matamoros et al., 2020 and others).

Lack of research on proprioceptive DAT impact on MDD and previous existing proof of psychosocial DAT effect supports the application of physically active programs such as DAT which leads to proprioception activation and potentially has positive effect in supporting muscle tension, biomechanical and viscoelastic properties and possibly having impact in quality of life.

## 2 Materials and methods

### 2.1 Participants

Two groups of participants were included in this study. A total of 14 adult participants were included in experimental group of this study and 14 adult participants were included in control group. All experimental group participants were diagnosed MDD and included in this case study between September 2022 to April 2024. MDD subjects were invited to participate in the study through a public call and by directly contacting psychiatric hospitals in Lithuania. However, due to MDD symptoms only 14 participants filled in the applications and were invited to participate. The inclusion criteria: (1) participants diagnosed MDD; (2) participants included in this study of working age; (3) never tried DAT; (4) agreed to participate into research and follow up. All participants were asked during DAT program to continue medication intake as prescribed in order not to change the conditions. Table 1 shows medication intake of MDD subjects. The composition of the control group in terms of age, gender and size was similar to the experimental group. Control group subjects had never been diagnosed of MDD, held as healthy. Prior to the study, all participants read and signed an informed consent form.

**Table 1:** Characteristics of study participants.

Gender	Age (yrs)	Currently used medications	Age when MDD diagnosis was set
Woman	45	Tetracyclic antidepressants	18 (27 yrs. MDD)
Woman	47	Tetracyclic antidepressants	40 (7 yrs. MDD)
Woman	31	Tetracyclic antidepressants	20 (11 yrs. MDD)
Man	40	Selective serotonin reuptake inhibitors	34 (6 yrs. MDD)
Woman	26	Selective serotonin reuptake inhibitors	12 (14 yrs. MDD)
Man	22	Tetracyclic antidepressants	15 (7 yrs. MDD)
Woman	48	Tetracyclic antidepressants, Atypical antipsychotics	25 (23 yrs. MDD)
Woman	51	Agomelatine	51 (1 yr. MDD)
Woman	58	Tetracyclic antidepressants	40 (18 yrs. MDD)
Woman	33	Selective serotonin reuptake inhibitors	22 (11 yrs. MDD)
Woman	61	Selective serotonin reuptake inhibitors	40 (21 yrs. MDD)
Woman	43	Agomelatine	36 (7 yrs. MDD)
Woman	42	Tetracyclic antidepressants	29 (13 yrs. MDD)
Woman	55	Selective serotonin reuptake inhibitors	46 (19 yrs. MDD)

### 2.2 Participant characteristics

A total of 14 adult MDD participants were included in experimental study. Table 1 provides the characteristics of the study participants. The mean age of the patients was 43 years (MIN = 22 MAX = 61), average MDD prevalence 13 yrs., two men and 12 women took part in the study. 35% of the participants had MDD diagnosis up to 10 yrs., 42.86% had MDD from 10 to 20 yrs., 21.43% had MDD over 20 yrs. All subjects of experimental group completed all dolphin assisted therapy sessions and 10 subjects filled in forms in one year. The age of 14 healthy subjects included into control group varied from 27 to 62 years old, there were 13 women and one man. Both group participants were right-handed.

In general, 14 Black Sea Bottlenose dolphins (*Tursiops truncatus ponticus – Latin*) were involved in the study. All participated dolphins were clinically healthy, the welfare was supervised by team of veterinarians and group of biologists, specialists in dolphin ethological behavior patterns. Dolphins interacted with MDD participants in special therapeutic pool marked by line with open escape if needed and accompanied by own biologist-dolphin trainer. To avoid any precautions, the behavior was constantly monitored, interacting with people was limited, breaks for supporting dolphin’s choice of communication pattern were made, swim with dolphins was not allowed. The study by the Ministry of Environment evaluated as low-risk, not needed special allowance.

### 2.3 Methods

The study involved MDD and healthy subjects. Since SF—36 scale has borderline parameters and was not applied for healthy subjects, a MyotonPro portable device requires to set the starting point in measuring muscle tension, biomechanical and viscoelastic properties. For this reason, healthy subjects were involved as a source of MyotonPro starting situation measurement borderline homogeneity and dynamics processing in MDD subjects.

We applied a two-week proprioceptive dolphin assisted activity program with 10 DAT sessions for experimental group with a two-days break after half activities. Description of proprioceptive application protocol in DAT sessions is described in Table 2. Each DAT session last for 30 min. Proprioceptive dolphin assisted activities varied from heavy muscle work to muscles and joints pressure which held in special therapeutic pool, adults wore wetsuits.

**Table 2:** Proprioceptive dolphin assisted therapy activities: description of intervention protocol.

Factor	Description	Method
Water	DAT lasts for 30 min. The sessions take place in the pool with water salinity 1.8%, the temperature—16 − 24° C.	Client moves against the resistance of water, their proprioceptors receive input. Buoyancy and upthrust gives proprioceptive receptors different stimulation than on land (Gjesing, 2002, 345).
Dolphin	Black Sea bottlenose dolphins (*Trusiops truncatus ponticus—lat.*) are the basic factor of the dolphin assisted therapy. The length of the dolphin’s body makes up to 3.5 m, the weight—300 kg approximately.	Dolphins are unique animals having the longest playing behavior in comparison to all other therapy animals. Therapy is provided for both enriching interactions. Dolphin assisted therapy is important as improving neurosensorymotor functions in participating client (Kreivinienė and Mockevičienė, 2020; Kreivinienė et al., 2021).
Wetsuit	During interaction with dolphins a participant must wear a 6 mm thickness wetsuit and wet shoes, which are extremely tight on the body.	Because of the wetsuit and high-water salinity, a participant receives a greater proprioceptive physical load in the water than usually swimming in public pools.
Activities	DAT program is designed for the best proprioceptive input while acting together with the dolphin.	Aqua Jogging (utilizing water resistance), lap swimming one hand/two hands handling rostrum of dolphin or object, treading, squats and lunges around the pool, floating and core work using water mat and noodle changing with supportive floating, floating on the back with dolphin support, strengthening core, joint compression provided by specialist.

No special applications or procedures were organised for control group subjects, they lived their ordinary life for 2 weeks and voluntarily took part in this research.

### 2.4 Data gathering

The SF—36 can be found in over 15.000 publications since it was standardized in 1990, around 1.500 of which are randomized control trials and over 1/3 of which are comparisons with alternate measures. The SF—36 is the only validated questionnaire in Lithuania which has been found to be reliable, valid, and responsive for a variety of medical diagnoses, reliable to measure correlation between psychological and physiological characteristics. Therefore, we used the Short Form Health SF-36 Survey scale for MDD participants of experimental group to investigate the quality of life of the participants. Participants were asked to answer the questions in eight subscales (functional capacity, functional limitation, pain, general health, vitality, social aspects, emotional aspects, and mental health). Participants of the experimental group were asked to fill in the SF-36 scale: (a) before dolphin assisted activities, (b) after two-week proprioceptive dolphin assisted activities, (c) in a year after two-week proprioceptive dolphin assisted activities. The score of SF-36 ranged between 0 (the worst quality of life) and 100 (the best quality of life).

The MyotonPro portable device was used for measuring muscle tension, biomechanical and viscoelastic properties. The Estonian MyotonPro portable device invented for non-invasive, objective measurement of mechanical muscles (Bailey et al., 2013). This device operates by giving a small mechanical impact to the tissue, the resulting oscillation of the tissues is recorded, and the parameters are calculated from curve (Ianieri et al., 2009). This generated oscillation in the measured muscle with a mechanical impact to the tissue (Ianieri et al., 2009) which was measured in particular muscle groups of MDD and healthy subjects as recommended. We applied MyotonPro equipped with a pointer that applies a perpendicular percussion to the biological tissue. The reliability and clinical application of this medical device was documented (see Bailey et al., 2013; Ianieri et al., 2009) as objective measure for parameters representing muscle tone, elasticity and stiffness. As Bailey et al. (2013) states, the device applies 10 mechanical impulses at one second intervals, producing damped oscillations, from which frequency (non-neural tone), stiffness and logarithmic decrement (elasticity) are objectively measured. In different MyotonPro-based studies it was proved that MyotonPro is reliable and valid measure method examining viscoelastic and biomechanic properties like vastus lateralis, rectus femoris, vastus medialis, biceps femoris, semitendinosus, quadriceps, and hamstrings (see Gacto-Sánchez et al., 2023), triceps brachii, biceps brachii, upper trapezius, and deltoid muscles (see Taş et al., 2023), dominant and non-dominant trapezius muscles (Liang et al., 2022). The systematic review evaluating the reliability of MyotonPro across various muscles for diagnostic purposes demonstrated promising reliability for diagnostic purposes across diverse patient populations and treatment (Lettner et al., 2024), therefore, it is stated that this device can be used not only as diagnostic but also as therapeutic intention evaluation method (Taş et al., 2023). Clinical MyotonPro device trials (see Shan et al., 2023) brought to the light that including evaluation the reliability and validity of using MyotonPRO to quantify the mechanical properties of the muscle-tendon unit through *in vivo* measurements and preliminary *in situ* measurements using formalin-fixed tissues, this device can be stated as a new tool for estimating strength with outstanding advantages: it is easy, time-efficient, adaptable, and highly manageable technology.

Subjects of experimental group were measured each day of participation: (a) before dolphin assisted activities and (b) after dolphin assisted activities. Control group participants were measured: (a) first day and (b) 2-weeks later.

MyotonPro was applied for each participant rested comfortably before measurement in the ambient-temperature room for 5 min to stabilise, surrounding was with minimal external disturbances. The assessment points were marked with a Medical Skin Marker on the largest cross-section of measured each muscle belly. The MyotonPro was applied to measure the upper (descending) part of the trapezius muscle (TM), lateral part of the deltoids muscle (DM), middle point of the flexor carpi radialis muscle (FCRM), middle point of the long head of the biceps brachii muscle (BM), the gastrocnemius muscle (plural gastrocnemii) (GM), and quadriceps femoris muscle (QFM). MyotonPro was obtained three times measurement series consisting of single measurements executed with an interval of 0.8 s and an average value was displayed. Participants were comfortably sitting on the chair with 90° leg-bend position and 90° degree elbow flexion position, participants were instructed to use this body position for full relaxation. When measuring, we kept the probe always perpendicular to the skin surface above the muscle being measured.

### 2.5 Ethical consideration

The study received ethical approval from the biomedical ethics committee of Lithuania no. BE 2-1-81.

## 3 Results

The measuring results of SF—36 scale showed statistically significant changes in 5 subscales out of 8 (Friedman’s test *p*-value less than 0.05) and in one more scale a partially significant change (*p* < 0.1). The physical activity that increased after dolphin therapy (from 68.5 to 85) remained the same in one year. There were no statistically significant changes in role limitations due to physical health. However, role limitations due to emotional problems and partially significant changes were recorded, the situation slightly worsened a year later, but there was no statistically significant deterioration. The non-parametric Wilcoxon test was used to compare two dependent samples measured by the MyotonPro. Although the improvement was recorded in almost all muscle groups, statistically significant changes were observed only in trapezius muscle md p right, measuring stiffness, relaxation and creep; trapezius md p left measuring frequency, stiffness, relaxation and decrement; and deltoids muscle left, measuring frequency, stiffness and relaxation, i.e., the *p*-values of Wilcoxon test are less than 0.05.

### 3.1 Results of quality of life

In order to determine the effect of dolphin therapy on a person’s physical and psychological state, the SF—36 questionnaire was applied 3 times: before starting dolphin therapy, immediately after therapy and one year later. Unfortunately, out of 14 individuals who participated in the therapy, only 10 agreed to participate in the study after a year.

Table 3 are the results of the Friedman test, and in Table 4 are the results of the Conover *Post Hoc* test. Statistically significant changes were recorded in 5 subscales out of 8 (Friedman’s test *p*-value less than 0.05) and in one more scale a partially significant change (*p* < 0.1) (Table 3).

**Table 3:** Descriptive statistics and Friedman test.

	Mean before therapy	Mean after therapy	Mean one year later	Kendall’s W	*p*
Physical functioning	68.5	85.0	85.0	0.586	**0.003**
Role limitations due to physical health	60.0	77.5	52.5	0.155	0.213
*Role limitations due to emotional problems*	40.0	66.667	60.0	0.261	*0.074*
Energy/fatigue	32.5	60.5	49.5	0.411	**0.016**
Emotional well-being	33.6	72.0	66.4	0.772	**<0.001**
Social functioning	40.25	70.25	59.75	0.32	**0.041**
Pain	51.5	79.0	49.25	0.458	**0.010**
General health	39.5	55.0	55.0	0.221	0.110

Statistically significant difference when Friedman’s test *p*-value is less than 0.05.

**Table 4:** Trapezius muscle right properties before and after DAT.

	Mean before	Mean after	W	*z*	*p*
Frequency	18.3	18.0	4470.000	1.658	0.097
Stiffness	350.9	336.9	5071.000	2.423	0.015
Decrement	1.19	1.18	4238.500	0.579	0.563
Relaxation	15.6	16.2	3024.500	−2.501	0.012
Creep	0.97	0.99	2807.500	−2.154	0.031

Statistically significant difference when Wilcoxon test *p*-value is less than 0.05.

The physical activity that increased after dolphin therapy (from 68.5 to 85) remained the same after one year. There are no statistically significant changes in role limitations due to physical health.

Role limitations due to emotional problems partially significant changes are recorded, i.e., improvement immediately after dolphin therapy, the situation slightly worsened a year later, but there is no statistically significant deterioration.

Immediately after the dolphin therapy, the energy increased, after a year it decreased slightly, but no statistically significant deterioration was recorded. The situation is similar with emotional well-being, the only difference in this case is that even after a year the situation is statistically significantly better. Similar conclusions can be made about social functioning. The results show a significant increase in pain immediately after therapy and return back of the pain ratio to a starting or even below after one year.

There are no statistically significant changes in general health estimation, nevertheless the mean increased from 39,5 to 55 and remained the same even after one year.

### 3.2 Results of muscle tension, biomechanical, and viscoelastic properties

Improvement in almost all muscle groups of experimental group were recorded. As it was expected, a statistically significant change in TM and DM parameters comparing muscle tension, biomechanical and viscoelastic properties before and after DAT. Change in TM right and left is seen mostly in decreased stiffness, increased relaxation, increased creep (see Tables 4, 5). Statistically insignificant, claiming betterment of TM right properties is decrement mean and TM both-sides frequency decrease. Statistically significant decrease TM left decrement.

**Table 5:** Trapezius muscle left properties before and after DAT.

	Mean before	Mean after	W	*z*	*p*
Frequency	19.1	18.8	4636.500	2.079	0.038
Stiffness	366.2	354.7	5143.000	2.782	0.005
Decrement	1.22	1.19	4821.000	2.359	0.018
Relaxation	14.9	15.4	3119.500	−2.145	0.032
Creep	0.93	0.95	3068.500	−1.745	0.081

DM right of subjects was measured as not having significant change, DM left statistically significant change was measured in decreased frequency, stiffness, increased relaxation and creep (Tables 6, 7). Analysing descriptives of the DM right and left, viscoelastic parameters of DM before N = 130, after N = 128. No statistically significant muscle properties were measured in biceps muscles (BM) left and right, flexor carpi radialis muscle (FCRM), the gastrocnemius muscle (plural gastrocnemii) (GM), and quadriceps femoris muscle (QFM) in subjects before and after DAT. However, insignificant change of muscle properties was measured.

**Table 6:** Properties of DM right in subjects before and after DAT.

	Mean before	Mean after	W	*z*	*p*
Frequency	14.36	14.38	3898.000	0.057	0.955
Stiffness	269.5	267.2	4205.000	0.823	0.411
Decrement	1.29	1.27	3.69.500	0.722	0.471
Relaxation	21.8	22.1	3867.000	−0.474	0.636
Creep	1.35	1.36	4014.500	−0.119	0.906

**Table 7:** Properties of DM left in subjects before and after DAT.

	Mean before	Mean after	W	*z*	*p*
Frequency	14.5	14.2	4802.500	2.498	0.013
Stiffness	274.6	261.8	5310.500	3.190	0.001
Decrement	1.35	1.34	4038.000	0.406	0.685
Relaxation	21.3	22.2	3003.500	−2.552	0.011
Creep	1.3	1.4	3121.500	−2.140	0.032

In order to gain more results, the results of muscle properties in subjects were compared with the results of healthy subjects. For the comparison nonparametric Mann–Whitney test was used. The results of the control group (healthy subjects) were statistically significantly different from the experimental group (MDD subjects) before DAT in TM. After two weeks, only 3 parameters on the right side and 4 on the left were significantly different, but these differences were already smaller, which indicates the improving condition of the patients (see results shown in Tables 8, 9).

**Table 8:** Results of TM right of experimental group control group.

	Group	Before DAT			After DAT		
		Mean	*W*	*p*	Mean	*W*	*p*
Frequency	Experimental	**19.1**	**148.000**	**0.006**	17.5	123.500	0.120
	Control	**16.2**			16.2		
Stiffness	Experimental	**369.9**	**148.000**	**0.006**	**334.3**	**133.000**	**0.043**
	Control	**284.1**			**284.1**		
Decrement	Experimental	**1.3**	**136.000**	**0.031**	1.2	123.500	0.120
	Control	**1.1**			1.1		
Relaxation	Experimental	**15.0**	**25.000**	**0.001**	**16.5**	**43.500**	**0.023**
	Control	**19.1**			**19.1**		
Creep	Experimental	**0.9**	**26.000**	**0.002**	**1.0**	**39.000**	**0.012**
	Control	**1.2**			**1.2**		

Statistically significant difference when Wilcoxon test *p*-value is less than 0.05.

**Table 9:** Results of TM left of experimental and control group.

	Group	Before DAT			After DAT		
		Mean	*W*	*p*	Mean	*W*	*p*
Frequency	Experimental	**19.8**	**166.500**	**<0.001**	19.3	127.000	0.085
	Control	**16.2**			16.2		
Stiffness	Experimental	**399.5**	**170.000**	**<0.001**	**352.4**	**135.000**	**0.035**
	Control	**278.9**			**278.9**		
Decrement	Experimental	**1.2**	**133.000**	**0.044**	**1.3**	**134.500**	**0.037**
	Control	**1.0**			**1.0**		
Relaxation	Experimental	**13.9**	**17.000**	**<0.001**	**15.8**	**48.500**	**0.041**
	Control	**18.8**			**18.8**		
Creep	Experimental	**0.9**	**20.500**	**<0.001**	**1.0**	**47.500**	**0.037**
	Control	**1.1**			1.1		

Statistically significant difference when Mann-Whitney test *p*-value is less than 0.05.

No statistically significant muscle properties were measured in biceps muscles (BM) left and right. Our research showed that measuring deltoideus muscle before starting the dolphin therapy, the results of the control and experimental groups does not differ statistically significant but after two weeks we can see that there is a statistically significant difference on the right side comparing 3 parameters, and on the left side—2, which shows an improvement in this muscle group as well (Tables 10, 11).

**Table 10:** Results of experimental and control group: DM right.

	Group	Before DAT			After DAT		
		Mean	*W*	*p*	Mean	*W*	*p*
Frequency	Experimental	14.8	69.000	0.296	**13.9**	**46.000**	**0.031**
	Control	15.6			**15.6**		
Stiffness	Experimental	282.3	84.000	0.752	246.6	52.000	0.062
	Control	284.2			284.2		
Decrement	Experimental	1.3	120.500	0.159	1.2	105.000	0.512
	Control	1.1			1.1		
Relaxation	Experimental	20.7	100.000	0.680	**23.6**	**137.000**	**0.027**
	Control	20.0			**20.0**		
Creep	Experimental	1.3	100.500	0.662	**1.4**	**134.000**	**0.039**
	Control	1.2			**1.2**		

Statistically significant difference when Mann-Whitney test *p*-value is less than 0.05.

**Table 11:** Results of experimental and control group: DM left.

	Group	Before DAT			After DAT		
		Mean	*W*	*p*	Mean	*W*	*p*
Frequency	Experimental	15.6	80.000	0.610	**14.1**	**51.500**	**0.048**
	Control	16.3			**16.3**		
Stiffness	Experimental	296.7	86.000	0.830	258.7	59.500	0.132
	Control	325.5			325.5		
Decrement	Experimental	1.3	109.500	0.382	**1.3**	**132.500**	**0.047**
	Control	1.1			**1.1**		
Relaxation	Experimental	19.6	87.000	0.865	22.9	123.500	0.120
	Control	19.2			19.2		
Creep	Experimental	1.2	89.500	0.961	1.4	125.000	0.103
	Control	1.2			1.2		

Statistically significant difference when Mann-Whitney test *p*-value is less than 0.05.

Our research in measuring gastrocnemius muscle (plural gastrocnemii) before DAT, the results of the control and experimental groups does not differ statistically significant but after two weeks we can see that there is a statistically significant difference on the right side comparing 2 parameters, and on the left side—3, which shows an improvement in this muscle group as well (Tables 12, 13).

**Table 12:** Results of experimental group before therapy and control group: GM right.

	Group	Before DAT			After DAT		
		Mean	*W*	*p*	Mean	*W*	*p*
Frequency	Experimental	13.7	75.000	0.452	12.8	76.500	0.496
	Control	13.7			13.7		
Stiffness	Experimental	257.6	64.000	0.202	**218.6**	**52.500**	**0.049**
	Control	254.1			**254.1**		
Decrement	Experimental	1.5	73.000	0.396	**1.2**	**43.000**	**0.021**
	Control	1.6			**1.6**		
Relaxation	Experimental	24.9	116.000	0.234	25.2	111.000	0.344
	Control	23.7			23.7		
Creep	Experimental	1.5	114.000	0.275	1.5	101.000	0.644
	Control	1.5			1.5		

Statistically significant difference when Mann-Whitney test *p*-value is less than 0.05.

**Table 13:** Results of experimental group before therapy and control group: GM left.

	Group	Before DAT			After DAT		
		Mean	*W*	*p*	Mean	*W*	*p*
Frequency	Experimental	14.4	65.500	0.225	13.0	54.500	0.080
	Control	14.3			14.3		
Stiffness	Experimental	279.3	64.500	0.207	**230.9**	**49.500**	**0.047**
	Control	264.0			**264.0**		
Decrement	Experimental	1.4	64.000	0.198	1.3	62.000	0.166
	Control	1.5			1.5		
Relaxation	Experimental	24.4	123.500	0.120	**25.4**	**134.500**	**0.037**
	Control	22.3			**22.3**		
Creep	Experimental	1.5	121.500	0.145	**1.5**	**134.000**	**0.039**
	Control	1.4			**1.4**		

Statistically significant difference when Mann-Whitney test *p*-value is less than 0.05.

## 4 Discussion

Our results indicate that there are statistically significant results in TM and DM muscle tension, biomechanical and viscoelastic properties after the 2-week proprioceptive DAT program. TM right and left in MDD subjects decreased in stiffness, increased in relaxation, and increased creep (only TM right). TM left in the two-week program statistically significant decreased decrement. DM right in two-week proprioceptive program with dolphins had significant change in muscle frequency and stiffness decrease, relaxation and creep increase. Comparing results of the TM experimental MDD group with the healthy group before DAT all muscle parameters – frequency, stiffness, decrement, relaxation and creep in both sides were statistically significant. Measurement comparing the change in groups after 2-weeks revealed that TM left remained statistically significant in all parameters, right – only stiffness. As far TM is upper trigger are the primary muscles responsible for neck pain and headaches also upper traps are also the most reactive muscles in your body to emotional stress, the findings reveal that 2-week DAT proprioceptive program had significant result in TM while measuring before and after in MDD group and before and after, showing that no significant difference was measured in MDD and healthy group. It means that MDD of TM in muscle tension, biomechanical and viscoelastic properties became close to healthy subjects.

Proprioception is mainly responsible for joint and body movements, positioning of body and its parts in space. The proprioceptors are responsible for continuous flow of sensations from muscle, joints and tendons. Therefore, this mechanism gives us information about the amount of force our muscles exerting, timing and rate of movements, and about muscle stretch (Smith, 2019). Recent theories about emotion and embodiment stress that proprioception is the core factor to socioemotional processes. In our case MDD is related to the processing and regulation of somatic states that are implicated in the construction of emotion (Riquelme et al., 2024). Studies document that the management of depression symptoms through exercise training is related to psychological, neuro-physiological, and neuro-developmental processes (Abdelbasset et al., 2020). Based on other studies Abdelbasset et al. (2020) argue that the protective effects of exercise against depression are centred on reinforcement, combining it with exercise-related neural regeneration and a growth agent. Riquelme et al. (2024) study reveals that position sense is important in different everyday situations including somatic and emotional responses. As it’s stated in Riquelme et al. (2024, cited in Hilber, 2022 and Balaban and Thayer, 2001) in this sense, brain structures such as the parabrachial nucleus or the cerebellum are involved in modulation of avoidance conditioning, anxiety and fear or adaptation to environmental stimuli on vestibular, somatic, visceral and exteroceptive sensory information converge. Therefore, measured positive muscle change in MDD subjects supports the suggestion of studies that physical exercise stimulates changes in the hypothalamic–pituitary–adrenal (HPA) axis, which normalizes the stress response, and changes in serotonergic neural activity in the dorsal raphe nucleus (Nabkasorn et al., 2006), which mediates the restoration of impaired behaviors in MDD (Greenwood et al., 2003). Physical exercise promotes synaptic plasticity by directly affecting synaptic structure and strength, as well as enhancing neurogenesis, vascular and metabolic functions (Abdelbasset et al., 2020). Depression involves boredom, reluctance and affects one’s thoughts, feelings and health, and well-being (Daneshvar et al., 2019). Proprioceptive DAT program proved by many studies as joyful unique program havingemotional proprioception mechanism (Finzi and Rosenthal, 2016). Emotional proprioception work both ways: MDD subjects were given proprioceptive input affecting lengthening of muscle spindles and muscle afferent firing and on the opposite – joyful facial expression from facial muscles affected their emotional state and made them judge pleasant proprioceptive stimuli as more positive. The encoding limb position and the creation of somatotopic maps generated positive behavior, social interactions and body empathy (Ackerley et al., 2017). Our study shows the proprioceptive DAT activities in MDD subjects decrease most reactive TM stiffness and relaxation parameters. However, contradict biomechanical increase in creep of TM and significant increase in pain while measuring subject with SF-36 can manifest of not adequate muscle rest in 48-h span and possible delayed-onset muscle soreness (DOMS) syndrome (Heiss et al., 2019) if continued for longer, same muscle group intensity period. Nevertheless, many studies manifest depression coherence with sedentary lifestyle (Daneshvar et al., 2019) and proprioceptive input, physical activity have reciprocal inverse relationship (Kumar and Jim, 2010; Edwards and Loprinzi, 2016; Blough and Loprinzi, 2018).

However, such objective methods as neuroimaging, electroencephalography or electromyography could evidence such a change which is limitedly applied in DAT studies because of different barriers. Separate non-parametric studies, such as Escobar et al. (2021) and Moreno Escobar et al. (2024) brings novel approaches about the both-sided brain effect by activating a patient’s particular brain regions as well as impacting the brainwaves of the dolphin. This is an important issue arising to analyse due to the spreading of differentiated animal-assisted practices with unconventional animal spices (see Suba-Bokodi et al., 2024; Baumgartner et al., 2024) under human care. Systemic meta-analysis (see Xiao et al., 2024) reveals the potential of different applied animal assisted therapies which helps to improve core symptoms in patients such as social communication, usage skills, irritability and others.

Counting a factor that MDD subjects because of low psychical activity had low level muscle tension for more than 2–29 years due to Henneman’s size principle of fixed-order motor-unit recruitment, we see mechanism similarity of the study of Garcia-Retortillo et al. (2023) who revealed that fundamental movement patterns require continuous skeletal muscle coordination, where muscle fibers with different timing of activation synchronize their dynamics across muscles with distinct functions it was uncovered that a network exhibits hierarchical organization (sub-networks/modules) with specific links and it was found network reorganization with fatigue where network modules follow distinct phase-space trajectories reflecting their functional role and adaptation to fatigue.

It can be stated that reactive muscles such as TM and DM showed statistically significant biomechanic and viscoelastic results both: before and after DAT of MDD subjects and in comparison to healthy subjects. We can make an assumption of this complex intermuscular network activates muscle fibres that facilitates movement and adapts to fatigue (Garcia-Retortillo et al., 2023) in MDD subjects. However, even though other muscle groups were insignificant statistically, the comparison of MDD subjects with healthy group showed important change proving this universality of the network system, which is independent on the specific movement. BM in comparison to MDD group before DAT muscle frequency (right MDD, before/after Mean = 12.157 / 11.446, healthy subjects = 11.446; left MDD, Mean = 11.5 / 11.729, healthy subjects = 11.185) remained without change, stiffness (right MDD, before / after Mean = 207.5 / 198.714, healthy subjects = 198.923; left MDD, Mean = 196.357 / 198.071, healthy subjects = 198.462) lowered in MDD group of BM right side and left side—increase, and relaxation (right MDD, before / after Mean = 26.714 / 27.536, healthy subjects = 28.131; left MDD, Mean = 28.85 / 28.686, healthy subjects = 29.362).

Statistically significant results of gastrocnemius muscle were got in comparison to healthy subjects. Muscle stiffness (*p* = 0.049) and decrement significantly decreased (*p* = 0.021) of GM right side and stiffness (*p* = 0.047) decreased, relaxation (*p* = 0.037) increased.

Our study showed that 2-week DAT proprioception program MDD subjects with right dominant side contribute to MDD patients to the body scheme and awareness to the external environment (Lane, 2002). This was proved by statistically significant change in fast-reacting T and D muscles while measuring before and after therapy, GM evaluating MDD muscle parameters with healthy subjects, and insignificant statistically however, important change in muscle properties development prognosis and it’s relationship to common proprioceptive body awareness and increase in QoL. In addition to our findings based on common proprioceptive and QoL change allows hypothesize that MDD subjects wearing wetsuits in high-salinity water experienced gavial insecurity crucially affecting vestibular system (Schaaf and Lane, 2009). Successful implications on vestibular system in MDD subjects could have a significant betterment in emotional status and future social functioning (Wilbarger and Wilbarger, 1991; Bundy and Murray, 2002). Vestibular system is composed of the following components: the semicircular canals and the otolith organs, the utricle and the saccule (Bundy and Murray, 2002). The otolith organs are responsible for static functions, the semicircular canals are responsible for dynamic functions. Vestibular sensory signal from the labyrinth of the inner ear provide direct information about rotations of the head and its orientation with respect to gravity. Semicircular canals and otolithic maculae are two types of vestibular transducer organs located within the labyrinth of the ear and respond to accelerations of the head in space. The three semicircular canals and the two otolithic maculae are stimulated by rotational and linear acceleration, respectively (Kreivinienė, 2016; Schaaf and Lane, 2009).

Interaction of the semicircular canals and the otolith organs allows directional detection of movement. Both these organs have receptor regions containing hair cells, the actual receptors throughout this system (Schaaf and Lane, 2009). The otolith organs are responsible for static functions, the semicircular canals are responsible for dynamic functions. Vestibular sensory signal from the labyrinth of the inner Activating otolith organs provides maintained compensation and stabilization of the head and upper trunk in upright position (Schaaf and Lane, 2009). The end result of this combination of semicircular canal and otolith receptors is that any movement of the head is detected; thus there is ongoing information to the central nervous system about head position in space and movement through space (Schaaf and Lane, 2009). The vestibular system becomes integrated with proprioception and vision via connections through the thalamus to the cerebral cortex (conscious awareness of body position in space), and the auditory system via a thalamic projection. In this way DAT proprioceptive program could have emotional modulation and betterment though vestibular input impact (Schaaf and Lane, 2009). Our research brought to the light that in comparison to healthy subjects’ main postural back muscles helping to maintain posture and supporting the body against gravity positively (QFM) and significantly (TM) changed as well as phasic muscles responsible for movement development positively (BBM) and significantly (DM) changed. Same idea was hypothesized in Muckelt et al. (2022) study that human body affected by postural insecurity affects most important postural and movement muscle groups. FCR muscle did not change as far proprioceptive DAT program was not involving imitative daily activities. As found in Brorsson et al. (2014) study writing with pen and scissors using mostly affects FCR. This improvement correlates with change in QoL significant change in physical activity, as far the GM as a complex muscle fundamental for walking, foot support, jumping, posture, slightly in knee function when quadriceps extend it. However, proprioceptive DAT gave MDD subjects active involvement in physical activities. Partly increased pain in MDD subjects related to the tibial nerve which is the reference nerve structure for the gastrocnemius muscle. Before dividing at the level of the upper corner of the popliteal fossa, in a tibial branch and a peroneal branch, these two nerves constitute the main trunk of the sciatic nerve; they are separated inside the sciatic by a fascial/adipose tissue or septum (Sladjana et al., 2008). Physical pain, especially chronic joint pain, limb pain and back pain is one of most often MDD symptoms (Trivedi, 2004). MDD subjects reported slightly increased pain just after DAT together with increased physical activity alongside emotional betterment. Even though GM betterment was statistically insignificant, remained reported same physical activity in a year impacted lowering pain and betterment in emotional health.

Our study thus confirms the results of other studies that proprioceptive DAT program as a kind of physical therapy manifests to have positive influence on QoL and perceived well-being in a wide range of patient populations including mental disorders (Kumar and Jim, 2010). It is not yet clear what is the mechanism of the effect of physical exercise on the mental state, but a list of assumptions and hypotheses is put forward. Tollár et al. (2020) based on their own and others’ studies, hypothesise that the improved perception of QoL may be related to exercise-induced increases in fitness. The results of our study also indicate a significant improvement in physical functioning and energy. Daneshvar et al., (2019) propose to apply the Pyrogen mechanism to explain the biochemical processes occurring in the brain of an exercising person with depression. Performing proprioceptive exercises in water, the physical load due to the wetsuit, and the program participant’s concentration on performing the exercises raise the body temperature, which has a positive effect on depression. In addition, the positive effect on depression is due to biochemical processes such as plasma catecholamine and monoamine release in the brain. During exercise, the level of the hormone endorphin in the blood increases (Daneshvar et al., 2019). Endorphin is an antidepressant hormone that creates vitality in the body. The most effective way to increase the level of endorphins in the body is to have a regular and exciting exercise (Cleare et al., 2015).

1 year after the 2-week DAT program, we asked proprioceptive DAT program participants to complete the SF—36 questionnaire again. The results of the study showed that the physical activity that increased after dolphin therapy (from 68.5 to 85) remained the same in one year. Energy (from 32.5 to 49.5), emotional well-being (from 33.6 to 66.4), social activity (from 40.25 to 59.75) indicators decreased compared to the ones immediately after the 2-week program, but did not return to the initial position, and remained significantly better. Pain came close to baseline, and was even lower (from 51.5 to 49.25). A survey conducted after 1 year also showed that participants rated their overall health equally well after 1 year as after 2-weeks DAT (from 39.5 to 55). Although the Friedman Test does not show statistical significance, the maintained stable indicator forms the assumption of complex changes in the daily life of the participants, which has not been included into the study. Moreover, maintaining the same level of physical activity indicates possible lifestyle changes that also have a direct positive effect on QoL.

Our study focuses on proprioceptive DAT effect and QoL to MDD subjects. Generally, DAT is evaluated controversial because of involvement of vulnerable groups, welfare of dolphins, validity limitations of small samples, placebo effects of exotic animals, family vacation, change of environment, and water (Marino and Lilienfeld, 2007; Fiksdal et al., 2012; Marine Connection: Protecting Dolphins and Whales Worldwide, 2009) stating that DAT can fleet improvements in mood (Marino and Lilienfeld, 2007). DAT effectiveness mechanism is only partly revealed as well as different methodologies underly such as “therapy.” By no means, DAT has several powerful characteristics of excitement and vitality: animal involvement and water. Furthermore, DAT that we targeted as an aquatic proprioceptive neuromuscular facilitation program shows even more significant changes in depression and improves QoL (Lee and Hwang, 2019). The improvements of different animal assisted therapy programs applied for patients witnessing promising results with long-term and follow-up data limitations (Xiao et al., 2024).

We did a follow up SF – 36 evaluation in a year, however, four MDD participants dropped out and the objective difference in MDD participants which we could not control was their intake or change of antidepressants as far they remain playing a prominent role in chronic conditions (Bonilla-Jaime et al., 2022). Half of MDD participants intake Tetracyclic antidepressants, others – newer class of antidepressants such as Selective Serotonin Reuptake Inhibitors or Agomelatine.

Our study suggests the novelty in DAT research compounding different factors and presenting DAT as proprioceptive program having positive effect for MDD patients. DAT is suggested to apply as complementary method to more traditional treatments (Nathanson et al., 1997). Different published studies same as this do not deny pharmacological treatment *per se*, but their results suggest that pharmacological treatment is not as effective. When supplemented with unconventional means, its health effects are significantly enhanced. For example, in 2019 and 2020, Abdelbasset and his team published the results of randomized controlled trial testing the effect of low to moderate-intensity continuous aerobic exercise on the depression status of various target groups of patients, for example patients with congestive heart failure or patients with diabetic neuropathy. The control groups in that extensive trial received conventional (pharmacological) interventions without any physical exercise. The findings showed the wide spectrum from non-significant changes in non-exercise (control group) (Abdelbasset et al., 2020) to a significant decrease in depression levels in exercise group only (ibid.) or both groups but twice greater reduction in exercise group when compared to a reduction in the control group (Abdelbasset and Alqahtani, 2019, Abdelbasset et al., 2019). Dong-Kyu and Tae-Yeun (2019) examined the effects of aquatic proprioceptive neuromuscular facilitation pattern exercise on various physiological and psychological metrics, including depression, in patients with chronic stroke. The experimental group performed aquatic proprioceptive neuromuscular facilitation pattern exercise. The control group performed ground proprioceptive neuromuscular facilitation pattern exercise. In a comparison between the two groups, the experimental group showed more significant positive changes in all measured metrics, including depression, than the control group.

Our study the same as in Abdelbasset et al. (2020) study involved MDD subjects undergoing traditional medical treatment. During participation in proprioceptive MDD program, they did not change medical treatment but no other interventions (as psychotherapy, etc.) were not applied. Our results indicate that two-weeks of proprioceptive dolphin assisted activities for adults with major depression have statistically significant change in better physical parameters. The quick significant change in TM and DM tend to ground DAT as stress relief, the other muscle groups change in comparison to healthy subjects allows us to hypothesize the intermuscular network activation leading to promising results. However QoL results in a year needs to be taken into account as could be affected by many side effects same as internal and external throughout the year: treatment, family situation changes, ending of COVID situation, etc.

## 5 Conclusion

This study investigated the therapeutic effect of proprioceptive DAT activities on muscle tension, biomechanical and viscoelastic properties and QoL in MDD subjects. Applying two-weeks of proprioceptive DAT activities for MDD adults had statistically significant and quick reactivity in TM and DM. The quick reactivity of TM revealed that DAT activities act as stress relief, and DM as relief of negative emotions, such as fear and anger. Even though other longer-reactivity muscle groups did not manifest statistical value before and after DAT, evaluating MDD muscle parameters with healthy subjects, important change in muscle properties development prognosis and its relationship to common proprioceptive body awareness and increase in QoL was set. The longer-reacting muscle groups such as GM, BM in comparison to muscle tension, biomechanical and viscoelastic properties in two-week DAT program significantly approached healthy subjects’ parameters. QoL parameters showed a long-lasting effect in keeping and sustaining physical activity during DAT and one year later. Overall, the study manifests that social life, pain, and health bettered in the two-week DAT program and one year later worsened but remained better if compared to initial status. Despite increasing number of studies evaluating proprioceptive exercise as an additional or direct method for managing depressive symptoms, our study is the first one attempting to measure a long-term effect. Main postural and back muscles and phasic muscles positively changed, also the pattern and strategy of DAT allows to hypothesize positive additional vestibular input. However, further causal investigation is necessary, particularly involving a broader range of participants with different MDD history, age and social factors. QoL parameters showed a long-lasting effect in keeping and sustaining physical activity during DAT and one year later. Overall, the study manifests that social life, pain, and health bettered in the two-week DAT program and one year later worsened but remained bettered if compared to initial status. However, further causal investigation is necessary, particularly involving a broader range of participants with different MDD history, age and social factors.

### 5.1 Limitations

The article only presents a case study with 14 patients there is no aim to generalize the results to the entire population. However, despite the exploratory and retrospective nature of this case study, it is acknowledged that these results must be discussed with added caution. The proprioceptive DAT program applied for MDD allows to make conclusions only as a case study understanding generalization limitations. Such DAT proprioceptive study is the first to review viscoelastic and biomechanic properties in MDD patients. Further studies expanding the number of researched subjects and follow up measurements is needed to generalize especially to viscoelastic and biomechanic change in muscle properties avoiding DOMS and deeper analysis of DAT as proprioceptive program alongside quality of life and MDD status betterment.

## Data Availability

The original contributions presented in the study are included in the article/Supplementary material, further inquiries can be directed to the corresponding author.
