# Supplementary material for: Therapeutic effect of proprioceptive dolphin assisted activities on health-related quality of life and muscle tension, biomechanical and viscoelastic properties in major depressive disorder adults: case analysis
Source: Front Hum Neurosci. 2025 Jan 14;18:1487293. doi: 10.3389/fnhum.2024.1487293 (PMC11772492; doi:10.3389/fnhum.2024.1487293)
Supplement: Supplementary file 1 [file Table_1.docx]

**DT data base**

| Object | Side | Frequency1 | Stiffness1 | Decrement1 | Relaxation1 | Creep1 | Frequency2 | Stiffness2 | Decrement2 | Relaxation2 | Creep2 |
| --- | --- | --- | --- | --- | --- | --- | --- | --- | --- | --- | --- |
| Trapez md p | Right | 15.5 | 242 | 0.84 | 18.5 | 1.08 | 16.6 | 327 | 1.25 | 16.9 | 1.07 |
| Trapez md p | Left | 19.1 | 364 | 1.04 | 13.7 | 0.84 | 24 | 436 | 1.7 | 11.3 | 0.71 |
| Deltoideus | Left | 12.8 | 226 | 1.17 | 22.6 | 1.36 | 12.4 | 212 | 1.25 | 26.3 | 1.57 |
| Deltoideus | Right | 12.7 | 217 | 1.1 | 22.2 | 1.3 | 12 | 182 | 0.87 | 28.6 | 1.7 |
| Bic Br c br | Left | 10.1 | 138 | 1.36 | 30.6 | 1.63 | 11.6 | 199 | 1.37 | 26.2 | 1.57 |
| Bic Br c br | Right | 10 | 136 | 1.35 | 30.5 | 1.62 | 11.3 | 199 | 1.37 | 27.6 | 1.66 |
| Gastr c m | Right | 11.9 | 187 | 0.94 | 25.3 | 1.47 | 13.6 | 240 | 1.12 | 23.5 | 1.44 |
| Gastr c m | Left | 11.3 | 217 | 1.3 | 26.8 | 1.62 | 14.7 | 266 | 1.01 | 20 | 1.23 |
| Trapez md p | Right | 17.3 | 320 | 1.2 | 17.8 | 1.1 | 16.2 | 331 | 1.37 | 17.5 | 1.09 |
| Trapez md p | Left | 19.8 | 445 | 1.16 | 12 | 0.76 | 14.8 | 269 | 1.27 | 21.9 | 1.35 |
| Deltoideus | Left | 16 | 319 | 1.22 | 17.5 | 1.09 | 12.7 | 228 | 1.14 | 23.8 | 1.45 |
| Deltoideus | Right | 15.3 | 295 | 1.38 | 19 | 1.18 | 12.6 | 221 | 1.06 | 28 | 1.72 |
| Bic Br c br | Left | 11.9 | 204 | 1.26 | 26.8 | 1.6 | 9.7 | 169 | 1.43 | 34.5 | 2.03 |
| Bic Br c br | Right | 11 | 187 | 1.42 | 28.1 | 1.64 | 10.1 | 154 | 1.33 | 32.3 | 1.81 |
| Gastr c m | Right | 10.9 | 194 | 1.63 | 28.7 | 1.69 | 11.8 | 150 | 0.65 | 29.6 | 1.68 |
| Gastr c m | Left | 10.4 | 175 | 1.25 | 32 | 1.89 | 11.8 | 201 | 1.19 | 28.4 | 1.73 |
| Trapez md p | Right | 15.5 | 293 | 1.18 | 18.7 | 1.16 | 15.3 | 284 | 1.17 | 18.5 | 1.14 |
| Trapez md p | Left | 18.9 | 407 | 1.54 | 14 | 0.91 | 16.9 | 336 | 1.32 | 16.6 | 1.06 |
| Deltoideus | Left | 13.4 | 214 | 0.96 | 23.1 | 1.4 | 13.5 | 213 | 1.31 | 25.4 | 1.56 |
| Deltoideus | Right | 14.2 | 237 | 0.99 | 21.2 | 1.29 | 13.9 | 210 | 1.14 | 22.8 | 1.37 |
| Bic Br c br | Left | 12.5 | 183 | 0.94 | 25.9 | 1.55 | 10 | 183 | 1.7 | 32 | 1.9 |
| Bic Br c br | Right | 12.3 | 201 | 1.26 | 27.4 | 1.67 | 10.5 | 166 | 1.14 | 31 | 1.81 |
| Gastr c m | Right | 13.5 | 213 | 1.23 | 24.3 | 1.45 | 15.1 | 239 | 0.94 | 20.6 | 1.25 |
| Gastr c m | Left | 12.8 | 217 | 1.04 | 25.8 | 1.57 | 16.5 | 267 | 1.04 | 19.3 | 1.2 |
| Trapez md p | Right | 16.1 | 327 | 1.62 | 17.9 | 1.13 | 16.1 | 340 | 1.4 | 18 | 1.14 |
| Trapez md p | Left | 15.9 | 340 | 1.44 | 18.3 | 1.16 | 17.5 | 332 | 1.08 | 16.5 | 1.04 |
| Deltoideus | Left | 15.8 | 317 | 1.31 | 19.3 | 1.22 | 16.3 | 301 | 1.31 | 18.9 | 1.17 |
| Deltoideus | Right | 15 | 299 | 1.16 | 19.5 | 1.21 | 13.9 | 219 | 0.79 | 23.1 | 1.37 |
| Bic Br c br | Left | 12.6 | 186 | 0.94 | 24.6 | 1.39 | 11.4 | 183 | 1.26 | 28.7 | 1.66 |
| Bic Br c br | Right | 13.7 | 209 | 0.92 | 23.3 | 1.37 | 11.5 | 173 | 1.19 | 28.2 | 1.61 |
| Gastr c m | Right | 15.7 | 279 | 1.72 | 19.8 | 1.22 | 16 | 256 | 0.85 | 19 | 1.11 |
| Gastr c m | Left | 19 | 402 | 1.21 | 13.4 | 0.83 | 16.9 | 276 | 0.73 | 17.1 | 0.99 |
| Trapez md p | Right | 18.1 | 364 | 1.36 | 15.6 | 1 | 18.2 | 369 | 1.43 | 15.4 | 0.97 |
| Trapez md p | Left | 19.2 | 370 | 1.62 | 15.5 | 0.99 | 17.1 | 337 | 1.45 | 16.6 | 1.04 |
| Deltoideus | Left | 13.6 | 279 | 1.72 | 23.8 | 1.51 | 14.3 | 302 | 1.76 | 24.1 | 1.54 |
| Deltoideus | Right | 13.1 | 285 | 1.73 | 26.3 | 1.69 | 13.1 | 280 | 1.8 | 26.5 | 1.68 |
| Bic Br c br | Left | 9.6 | 193 | 1.6 | 35.4 | 2.15 | 9.8 | 183 | 1.47 | 34.2 | 2.06 |
| Bic Br c br | Right | 12.3 | 196 | 1.22 | 27.7 | 1.68 | 10 | 196 | 1.62 | 32.9 | 1.99 |
| Gastr c m | Right | 13.7 | 234 | 2.01 | 26.8 | 1.67 | 12.3 | 245 | 1.68 | 24.9 | 1.53 |
| Gastr c m | Left | 11.6 | 207 | 1.5 | 27 | 1.61 | 11.8 | 255 | 2.07 | 26.2 | 1.61 |
| Trapez md p | Right | 17.5 | 311 | 1.22 | 17.4 | 1.08 | 19.3 | 353 | 0.97 | 14.2 | 0.87 |
| Trapez md p | Left | 18.8 | 358 | 1.18 | 15.4 | 0.97 | 17.9 | 327 | 1.04 | 15.9 | 0.98 |
| Deltoideus | Left | 17.1 | 329 | 1.13 | 16.7 | 1.04 | 13.5 | 218 | 1.06 | 23.9 | 1.45 |
| Deltoideus | Right | 16 | 284 | 1.03 | 18.7 | 1.16 | 14.6 | 250 | 1.2 | 21.4 | 1.33 |
| Bic Br c br | Left | 9.7 | 143 | 1.05 | 36.2 | 2.07 | 10.6 | 164 | 1.9 | 31.4 | 1.83 |
| Bic Br c br | Right | 10 | 154 | 1.01 | 32.9 | 1.9 | 10.7 | 171 | 1 | 30.7 | 1.84 |
| Gastr c m | Right | 27.1 | 679 | 1.16 | 7.7 | 0.5 | 11.8 | 173 | 1.01 | 28.2 | 1.61 |
| Gastr c m | Left | 31.9 | 784 | 1.18 | 6.6 | 0.44 | 11.1 | 194 | 1.23 | 29.9 | 1.81 |
| Trapez md p | Right | 19.6 | 391 | 1.13 | 13.5 | 0.85 | 19.4 | 380 | 1.03 | 13.8 | 0.86 |
| Trapez md p | Left | 23.1 | 513 | 0.94 | 10 | 0.63 | 16.9 | 295 | 0.91 | 17.4 | 1.07 |
| Deltoideus | Left | 15.6 | 283 | 0.98 | 17.7 | 1.07 | 13 | 229 | 1.14 | 25.1 | 1.54 |
| Deltoideus | Right | 17.9 | 382 | 1.16 | 14 | 0.87 | 13.2 | 246 | 1.2 | 25 | 1.57 |
| Bic Br c br | Left | 10.9 | 204 | 1.58 | 27.7 | 1.69 | 11.7 | 205 | 1.39 | 26.9 | 1.64 |
| Bic Br c br | Right | 13 | 208 | 1.92 | 26.8 | 1.64 | 12.2 | 194 | 1.13 | 25.7 | 1.55 |
| Gastr c m | Right | 10.8 | 181 | 1.23 | 31.2 | 1.85 | 11.5 | 186 | 1.12 | 27.4 | 1.61 |
| Gastr c m | Left | 12.1 | 205 | 1.25 | 27.4 | 1.66 | 11.5 | 214 | 1.42 | 28.6 | 1.76 |
| Trapez md p | Right | 25 | 567 | 1.32 | 9.2 | 0.6 | 22.4 | 486 | 1.25 | 11.2 | 0.72 |
| Trapez md p | Left | 23.3 | 482 | 1.1 | 10.8 | 0.69 | 20.6 | 390 | 1.5 | 14.1 | 0.87 |
| Deltoideus | Left | 17.8 | 340 | 1.47 | 16 | 1.01 | 13.5 | 269 | 1.8 | 26.5 | 1.65 |
| Deltoideus | Right | 14.7 | 278 | 1.49 | 19.8 | 1.24 | 14.5 | 223 | 1.49 | 24.5 | 1.42 |
| Bic Br c br | Left | 12.6 | 233 | 1.99 | 28.6 | 1.79 | 17 | 254 | 1.87 | 22 | 1.33 |
| Bic Br c br | Right | 12.7 | 250 | 2.28 | 27.1 | 1.72 | 17.3 | 272 | 2.27 | 20 | 1.21 |
| Gastr c m | Right | 12.1 | 247 | 2.42 | 30.3 | 1.9 | 14.1 | 223 | 1.26 | 24.4 | 1.5 |
| Gastr c m | Left | 12.4 | 274 | 2.82 | 28.5 | 1.81 | 13.4 | 212 | 1.16 | 25.6 | 1.58 |
| Trapez md p | Right | 17.6 | 362 | 1.16 | 15.5 | 0.97 | 13.7 | 182 | 0.86 | 20.1 | 1.03 |
| Trapez md p | Left | 16.6 | 351 | 1.36 | 16.5 | 1.06 | 14.5 | 226 | 0.81 | 19.7 | 1.12 |
| Deltoideus | Left | 13.5 | 182 | 0.89 | 23.2 | 1.3 | 12.8 | 222 | 1.09 | 22 | 1.29 |
| Deltoideus | Right | 12.4 | 185 | 0.93 | 28.7 | 1.74 | 13.1 | 224 | 0.94 | 19.7 | 1.12 |
| Bic Br c br | Left | 11.9 | 205 | 1.48 | 24.5 | 1.46 | 11.9 | 154 | 0.91 | 26.6 | 1.44 |
| Bic Br c br | Right | 12.2 | 229 | 1.72 | 24.1 | 1.48 | 12 | 189 | 1.05 | 25.6 | 1.48 |
| Gastr c m | Right | 15.2 | 288 | 1.12 | 18.8 | 1.17 | 12.6 | 211 | 0.96 | 25.3 | 1.51 |
| Gastr c m | Left | 16.4 | 296 | 1.07 | 18.7 | 1.18 | 11.5 | 181 | 0.9 | 29 | 1.7 |
| Trapez md p | Right | 23.3 | 483 | 1.99 | 11.6 | 0.75 | 20.2 | 416 | 1.39 | 12.3 | 0.76 |
| Trapez md p | Left | 20.7 | 418 | 1.55 | 13.7 | 0.87 | 35.6 | 615 | 3 | 7.3 | 0.47 |
| Deltoideus | Left | 21 | 463 | 1.53 | 11.8 | 0.75 | 15.7 | 296 | 1.21 | 18.2 | 1.12 |
| Deltoideus | Right | 17.2 | 333 | 1.38 | 17.3 | 1.08 | 15.2 | 280 | 1.19 | 20.2 | 1.25 |
| Bic Br c br | Left | 13.2 | 220 | 1.75 | 27.1 | 1.63 | 11.2 | 203 | 1.81 | 29.3 | 1.76 |
| Bic Br c br | Right | 14.2 | 258 | 2.39 | 24 | 1.46 | 11 | 208 | 1.96 | 27.9 | 1.66 |
| Gastr c m | Right | 13.1 | 233 | 1.51 | 25.2 | 1.53 | 14.3 | 286 | 1.65 | 19.4 | 1.2 |
| Gastr c m | Left | 15.4 | 283 | 1.86 | 21.1 | 1.31 | 12.7 | 234 | 1.42 | 26.9 | 1.67 |
| Trapez md p | Right | 20.7 | 420 | 1.07 | 12.8 | 0.81 | 17.9 | 342 | 1.27 | 16.3 | 1.02 |
| Trapez md p | Left | 23.7 | 499 | 1.16 | 10.6 | 0.68 | 18.6 | 357 | 1.35 | 15.6 | 0.99 |
| Deltoideus | Left | 11.9 | 269 | 1.88 | 28.5 | 1.8 | 15.3 | 305 | 1.63 | 19.8 | 1.28 |
| Deltoideus | Right | 11.9 | 269 | 1.75 | 24.9 | 1.55 | 13.2 | 287 | 1.79 | 26.5 | 1.68 |
| Bic Br c br | Left | 10.4 | 200 | 1.94 | 31.1 | 1.88 | 10.8 | 210 | 1.89 | 30.1 | 1.82 |
| Bic Br c br | Right | 11 | 207 | 1.83 | 29.6 | 1.8 | 11 | 220 | 2.22 | 28 | 1.7 |
| Gastr c m | Right | 10.9 | 195 | 1.34 | 29.8 | 1.79 | 11 | 209 | 1.62 | 28.6 | 1.7 |
| Gastr c m | Left | 11 | 198 | 1.2 | 29.3 | 1.77 | 11.2 | 216 | 1.5 | 28.3 | 1.69 |
| Trapez md p | Right | 22.7 | 462 | 1.23 | 11.3 | 0.73 | 19 | 344 | 1.05 | 15.4 | 0.95 |
| Trapez md p | Left | 20.8 | 414 | 1.26 | 13.5 | 0.87 | 18.6 | 338 | 1.22 | 15.5 | 0.96 |
| Deltoideus | Left | 12.8 | 259 | 1.51 | 24.1 | 1.51 | 13.2 | 272 | 1.52 | 25.4 | 1.61 |
| Deltoideus | Right | 12.7 | 259 | 1.58 | 23.7 | 1.48 | 13.8 | 252 | 1.21 | 24.5 | 1.54 |
| Bic Br c br | Left | 11 | 210 | 1.76 | 29.6 | 1.83 | 12.5 | 211 | 1.92 | 27.3 | 1.66 |
| Bic Br c br | Right | 11.8 | 206 | 1.52 | 27.3 | 1.65 | 11.3 | 202 | 1.91 | 28.4 | 1.72 |
| Gastr c m | Right | 11.5 | 227 | 1.59 | 27.5 | 1.71 | 11.2 | 224 | 1.79 | 28.2 | 1.73 |
| Gastr c m | Left | 12.1 | 227 | 1.7 | 29.2 | 1.82 | 11.6 | 232 | 1.86 | 27.9 | 1.71 |
| Trapez md p | Right | 19.7 | 364 | 1.07 | 14.1 | 0.88 | 17.6 | 323 | 1.15 | 17.3 | 1.08 |
| Trapez md p | Left | 20.1 | 354 | 1.14 | 14.2 | 0.88 | 21.4 | 439 | 1.11 | 12.3 | 0.78 |
| Deltoideus | Left | 18.6 | 361 | 1.22 | 15.5 | 0.99 | 16.8 | 316 | 1.36 | 18.6 | 1.17 |
| Deltoideus | Right | 17.6 | 332 | 1.19 | 17 | 1.08 | 15.6 | 291 | 1.39 | 20.9 | 1.32 |
| Bic Br c br | Left | 13.4 | 249 | 1.98 | 25.6 | 1.62 | 14 | 268 | 1.36 | 23.3 | 1.45 |
| Bic Br c br | Right | 13.3 | 231 | 1.28 | 25.2 | 1.57 | 13.7 | 244 | 1.23 | 21.6 | 1.32 |
| Gastr c m | Right | 12.2 | 206 | 1.07 | 26.9 | 1.65 | 11.8 | 210 | 1.24 | 28 | 1.69 |
| Gastr c m | Left | 11.8 | 187 | 1.01 | 30.3 | 1.85 | 12.9 | 231 | 1.26 | 25.8 | 1.58 |
| Trapez md p | Right | 19.1 | 272 | 1.39 | 16.7 | 0.97 | 13.6 | 203 | 1.04 | 23.9 | 1.41 |
| Trapez md p | Left | 17 | 278 | 0.68 | 16.6 | 0.99 | 15.3 | 236 | 1.12 | 20.4 | 1.2 |
| Deltoideus | Left | 18.5 | 313 | 0.82 | 14.7 | 0.89 | 14.2 | 239 | 1.19 | 22.1 | 1.34 |
| Deltoideus | Right | 16.5 | 297 | 1.18 | 17.2 | 1.07 | 16 | 288 | 0.95 | 18.4 | 1.13 |
| Bic Br c br | Left | 11.2 | 181 | 1.4 | 30.2 | 1.82 | 12 | 187 | 1.5 | 29.1 | 1.73 |
| Bic Br c br | Right | 12.7 | 233 | 1.71 | 20 | 1.17 | 13.2 | 194 | 1.72 | 25.6 | 1.5 |
| Gastr c m | Right | 12.9 | 243 | 1.56 | 25.6 | 1.58 | 12.7 | 209 | 1.18 | 26 | 1.57 |
| Gastr c m | Left | 13.3 | 238 | 1.28 | 25 | 1.57 | 13.7 | 253 | 1.41 | 23.2 | 1.43 |

**SF data base**

| Before | | | | | | | | After | | | | | | | |
| --- | --- | --- | --- | --- | --- | --- | --- | --- | --- | --- | --- | --- | --- | --- | --- |
| Physical functioning | Role limitations due to physical health | Role limitations due to emotional problems | Energy/fatigue | Emotional well-being | Social functioning | Pain | General health | Physical functioning | Role limitations due to physical health | Role limitations due to emotional problems | Energy/fatigue | Emotional well-being | Social functioning | Pain | General health |
| 80,0 | 50,0 | 100,0 | 35,0 | 40,0 | 57,5 | 22,5 | 45,0 | 100,0 | 100,0 | 100,0 | 80,0 | 72,0 | 100,0 | 100,0 | 100,0 |
| 30,0 | 25,0 | 0,0 | 35,0 | 52,0 | 67,5 | 45,0 | 30,0 | 45,0 | 50,0 | 100,0 | 80,0 | 88,0 | 100,0 | 45,0 | 50,0 |
| 85,0 | 50,0 | 0,0 | 20,0 | 36,0 | 35,0 | 22,5 | 30,0 | 95,0 | 100,0 | 100,0 | 75,0 | 88,0 | 57,5 | 100,0 | 40,0 |
| 85,0 | 25,0 | 66,7 | 65,0 | 56,0 | 55,0 | 100,0 | 75,0 | 90,0 | 50,0 | 100,0 | 70,0 | 84,0 | 80,0 | 100,0 | 85,0 |
| 85,0 | 0,0 | 33,3 | 75,0 | 48,0 | 55,0 | 57,5 | 50,0 | 85,0 | 25,0 | 33,3 | 55,0 | 52,0 | 65,0 | 57,5 | 55,0 |
| 70,0 | 100,0 | 66,7 | 55,0 | 44,0 | 65,0 | 35,0 | 40,0 | 70,0 | 75,0 | 66,7 | 70,0 | 56,0 | 55,0 | 42,5 | 40,0 |
| 55,0 | 25,0 | 33,3 | 20,0 | 24,0 | 22,5 | 32,5 | 25,0 | 95,0 | 75,0 | 66,7 | 65,0 | 80,0 | 65,0 | 67,5 | 55,0 |
| 40,0 | 25,0 | 33,3 | 20,0 | 32,0 | 32,5 | 67,5 | 45,0 | 85,0 | 75,0 | 100,0 | 80,0 | 80,0 | 100,0 | 100,0 | 65,0 |
| 50,0 | 25,0 | 0,0 | 25,0 | 32,0 | 32,5 | 67,5 | 30,0 | 75,0 | 50,0 | 66,7 | 75,0 | 72,0 | 77,5 | 100,0 | 60,0 |
| 95,0 | 100,0 | 66,7 | 45,0 | 56,0 | 55,0 | 100,0 | 90,0 | 95,0 | 100,0 | 66,7 | 55,0 | 68,0 | 77,5 | 100,0 | 90,0 |
| 45,0 | 25,0 | 0,0 | 10,0 | 24,0 | 22,5 | 22,5 | 20,0 | 90,0 | 75,0 | 33,3 | 80,0 | 96,0 | 87,5 | 57,5 | 30,0 |
| 70,0 | 100,0 | 0,0 | 20,0 | 8,0 | 10,0 | 67,5 | 20,0 | 90,0 | 100,0 | 33,3 | 60,0 | 52,0 | 55,0 | 77,5 | 45,0 |
| 45,0 | 100,0 | 33,3 | 15,0 | 36,0 | 45,0 | 67,5 | 40,0 | 65,0 | 50,0 | 66,7 | 70,0 | 80,0 | 90,0 | 100,0 | 60,0 |
| 55,0 | 25,0 | 33,3 | 15,0 | 12,0 | 35,0 | 45,0 | 10,0 | 60,0 | 50,0 | 33,3 | 25,0 | 44,0 | 35,0 | 45,0 | 5,0 |
